# Cyclosporine treatment for steroid-resistant nephrosis complicated with acquired hemophilia A: a case report

**DOI:** 10.1097/MS9.0000000000000353

**Published:** 2023-04-03

**Authors:** Mohammad Alsultan, Hiba Jomaa, Arwa Shukri, Fatima Hajij, Qussai Hassan

**Affiliations:** a Department of Nephrology; b Department of Hematology; c Department of Internal Medicine; d Department of Internal Medicine; e Prof and chief of Nephrology Department, Damascus University- Faculty of medicine, Al Assad University Hospital, Damascus, Syria

**Keywords:** acquired hemophilia A (AHA), anti-factor VIII, cyclosporine, nephrotic syndrome (NS), steroids- resistant nephrosis (SRN)

## Abstract

Acquired hemophilia refers to the development of a clotting factor deficiency typically, when autoantibodies are produced against coagulation factor and tend to be in the elderly and rare in children.

**Case report:**

A 12-year-old girl with steroid-resistant nephrosis (SRN) was admitted complaining of pain in her right leg and an ultrasound showed a hematoma in her right calf. The coagulation profile revealed partial thromboplastin time prolongation and high titers (156 BU) of anti-factor VIII inhibitors were observed. Where half of the patients with antifactor VIII inhibitors were associated with underlying disorders, additional tests were performed that rule out secondary causes. This patient with long-standing SRN, who was on a maintenance dose of prednisone for six years, was complicated with acquired hemophilia A (AHA). In different to the last recommendations of AHA treatment, we preferred using cyclosporine; which is considered the initial second-line therapy for children with SRN. Complete remission was achieved of both disorders after a month with no recurrence of nephrosis or bleeding events.

**Conclusion:**

To our knowledge, nephrotic syndrome with AHA was reported in only three patients, two cases after remission and one during a relapse but no one were treated with cyclosporine. The authors encountered the first case of cyclosporine treatment for AHA in a patient with SRN. This study supports the use of cyclosporine to treat AHA, especially with nephrosis.

## Introduction

Highlights

- A patient with steroids resistant nephrosis was complicated with acquired hemophilia A.
- A high titer of anti-FVIII (156 BU) was calibrated, that had not been reported in this value before.
- We encountered the first case of cyclosporine treatment for acquired hemophilia A complicated with steroids resistant nephrosis.
- Adding cyclosporine induced quickly remission; after a month, of both diseases.

Acquired hemophilia refers to the development of a clotting factor deficiency that was not present at birth, typically, when autoantibodies are produced against the coagulation factor^1^. Acquired hemophilia A (AHA) (acquired inhibitors against factor VIII) is the most common and tends to be in the elderly and rare in children^1^. Half of patients are associated with underlying disorders such as autoimmune, malignancy, and drugs^1^. Several approaches were used for treating AHA, such as steroids alone; which is still the mainstay of the treatment by using alone (remission in 30% of cases), or combining with cyclophosphamide (remission in 60–70% of cases)^1^–^3^.

Since the vast majority of young children with nephrotic syndrome (NS) have minimal change disease (MCD), they do not undergo a biopsy at presentation unless they have features suggesting other diagnosis and are highly (>95%) to experience a complete remission with steroid therapy^4^. However, the treatment of the MCD children who are resistant to or dependent on corticosteroids remains a challenge, despite the several therapeutic options available^4^.

This case reported a young girl with AHA associated with steroid-resistant nephrosis (SRN), who was on a maintenance dose of prednisone. Receiving cyclosporine with steroid achieved complete remission of both disorders. This case report is according with the Declaration of Helsinki and in line with the Surgical CAse REport (SCARE) Guidelines^5^.

## Case report

A 12-year-old girl was admitted to our Nephrology Department due to pain in her right lower leg that began 3 days ago. Her past medical history consisted of nephrosis, which was diagnosed and treated in her town 6 years ago, and is still on a maintenance dose of prednisone 5 mg/d. Also, urine tests (24 h urine protein and a random protein to creatinine ratio) during the treatment period correspond with SRN.

**Figure 1:**
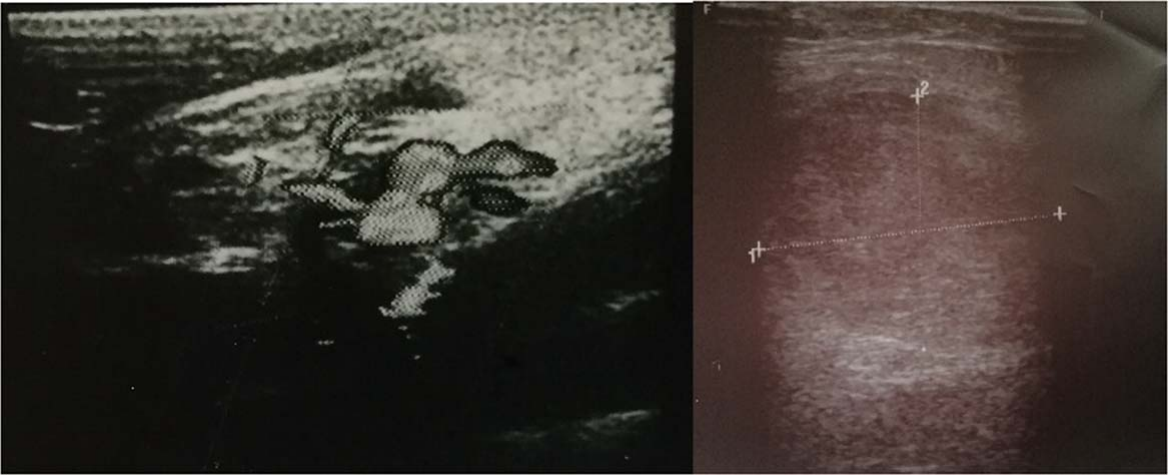
Ultrasound revealed an accumulation of hematoma in the right calf.

On physical examination: temperature 37C, blood pressure 100/60 mmHg, pulse rate 74 /min, and respiratory rate 14 /min. She had swelling in her right calf and movement restriction with no redness or local warmth and no history of trauma. Ultrasonography showed a hematoma in the right calf (Fig. 1).

The first decision to reevaluate NS was a kidney biopsy, but a calf hematoma obligated hematologic evaluation (Table 1), which showed partial thromboplastin time prolongation. A mixing test was done and compatible with coagulation factors antibodies (Table 1)^6^. Anti-factor VIII inhibitors (Anti-FVIII) were positive with high titers (156 BU) and lupus anticoagulant was negative. As mentioned before^1^, additional tests to rule out secondary causes were performed and all came back negative (Table 1). A full-body computed tomography was negative for lymphadenopathy and malignancy. Due to NS, additional tests were performed; protein C, protein S, and antithrombin III, and all within normal limits.

**Table 1:** Laboratory tests

On admission					
WBCs	11.8	HDL	67	Cr /24 h	832 mg
Hb	12.7	PT	81%	HBS Ag	Neg
HT	32	INR	1.15	Anti HCV Ab	Neg
PLT	372	aPTT	79	HIV antigen	Neg
Ur	9	ESR	99	ANA	Neg
Cr	0.4	CRP	0.9	RF	Neg
TP	4.8	FVIII Act	0.5%	anti-CCP	Neg
Alb	2.2	FVIII Inh	>156	ACL	Neg
Chol	194	Urine volume	1800 ml	anti B2GP	Neg
LDL	116	Protein /24 h	8116 mg	C3	114
TG	141	Alb/24 h	5760 mg	C4	20
Mixing Test					
	Before		Directly after		1 h after
PT	67%		88%		90%
INR	1.32		1.08		1.07
aPTT	79.1		46		64

ACL, anticardiolipin antibody; ALB, albumin; ANA, antinuclear antibody; anti B2GP, anti b2 glycoprotein antibody; Anti HCV Ab, anti-Hepatitis C Antibody; anti-CCP, cyclic citrullinated peptide antibodies; aPTT, activated partial thromboplastin time; C3-4, complements; Chol, cholesterol; Cr, creatinine; ESR, erythrocyte sedimentation rate; FVIII Act, factor VIII activity; HB, hemoglobin; HBS Ag, Hepatitis B Antigen; HDL, high-density lipoprotein; HT, hematocrit; Inh, inhibitor; INR, international normalized ratio; LDL, low-density lipoprotein; PLT, platelets; PT, prothrombin time; RF, rheumatoid factor; TG, Triglycerides; TP, total protein; Ur, urea; WBC, white blood count.

Since calcineurin inhibitors (CNI) such as cyclosporine is considered the initial second-line therapy for children with SRN^7^, we started cyclosporine with prednisone. Initial prednisone dose was 40 mg/d and the tapered and discontinued after 6 months. Cyclosporine was started at 100 mg/ twice daily with the previous dose for the first 2 months and then tapered slowly to a maintenance dose of 100 mg/d after the first year. Activated partial thromboplastin time prolongation and proteinuria returned to normal limits after a month. The hematoma was resolved after 2 months with no recurrence of nephrosis or bleeding events. Monitoring tests are shown in Table 2 and the patient is currently on a tapering regimen to complete the 2-year course of the treatment.

**Table 2:** Monitoring test during the course of treatment

Months after treatment	aPTT (sec)*	FVIII inhibitor (BU)	Proteinuria
1	45	—	112 mg /24 h urine
2	40	—	†ACR 0.14
4	51	35	ACR 0.39
6	49	—	ACR 0.48
8	44	—	ACR 0.35
10	41	—	ACR 0.32
12	35	—	ACR 0.31
14	32	FVIII act 68%	ALB 4.7 mg\dl ACR 0.2 51 mg /24 h urine

* aPTT; normal rang (21–35 s).

† ACR; albumin to creatinine ratio in random urine sample ( normal range up to 0.2).

## Discussion

This report described AHA with a long-standing SRN. A high titer of anti-FVIII was calibrated, and adding cyclosporine resulted in remission of both disorders, which has not been reported in the literature.

To our knowledge, NS complicated with AHA was reported in only three patients^2^,^3^,^8^. The first described a 2-year-old boy with NS complicated with AHA before the completion of the prednisolone administration^2^. Thereafter, the patient was started on treatment with three courses of steroid pulse therapy (methylprednisolone), which was associated with anti-FVIII remission but switching to cyclosporine after a frequent recurrence of nephrosis without recurrence of hemophilia^2^. The second described a 39-year-old male with MCD that was complicated with AHA. The remission had been reported after treatment with prednisolone and cyclophosphamide^3^. The last case of AHA in a 74-year-old male with relapsing MCD was given prednisolone and obtained remission of both diseases after 6 weeks^8^. In the current case, a patient with SRN, who was on a maintenance dose of prednisone, was complicated with anti-FVIII. Thereafter, adding cyclosporine induced quickly remission; after a month, of both diseases.

Prednisone and activated prothrombin complex concentrates was effective for treating high titers of anti-FVIII (the highest was 155 BU) in seven of eight patients without underlying disorders^9^. But this report described the incidence of AHA with NS and recorded a higher titer of anti-FVIII (156 BU), which had not been reported in this value before.

Another report reviewed two consecutive patients with AHA with a successful treatment with cyclosporine 100 mg twice daily^10^. These remissions have been maintained for 20 and 24 months suggesting further investigation of cyclosporine in AHA^10^. In our patient, the cyclosporine dose was resembled and supported its use in AHA.

Several drugs were implicated in AHA, which was reported in a 4-year-old boy after oral penicillin and treated with a high dose of recombinant FVIII^11^. This study suggested more quickly resolving of inhibitors in childhood than adults, and resolution might not require immunosuppressive agents^11^. Our patient achieved a normal activated partial thromboplastin time after a month of starting treatment, however, this case supported the essential role of immunosuppression, and a long course might be needed for high titers of antibodies.

The last recommendations for treatment of AHA were suggested steroids along with rituximab or cytotoxic agents (cyclophosphamide or mycophenolate mofetil) as the first-line therapy for FVIII <1 IU/dl or inhibitor titer greater than 20 BU^12^. Since CNI is considered to be more efficacy based on a relatively higher value on data suggesting that CNIs are more likely to induce remission than cyclophosphamide, mycophenolate mofetil, or rituximab in the treatment of children with SRN^7^, we preferred its use in this patient, which achieved the desired goal.

It is clear that this case only gives an option in AHA treatment but future researches are needed to prove this benefit in AHA treatment and comparing with other immunosuppression in the last recommendations.

## Conclusion

We encountered the first case of cyclosporine treatment for AHA in a patient with steroids resistant nephrosis. This study suggest cyclosporine as a novel option for treat AHA, especially with nephrosis.

## Ethical approval

Written informed consent was obtained from the father of patient for publication of this case report and accompanying images, in line with local ethical approval requirements and in accordance with the Helsinki Declaration.

## Consent

Written informed consent was obtained from the father of the patient for publication of this case report and accompanying images. A copy of the written consent is available for review by the Editor-in-Chief of this journal on request.

## Sources of funding

This research did not receive any specific Grant from funding agencies in the public, commercial, or not-for-profit sectors.

## Author contributions

Dr M.A.: writes the manuscript, literature search, submit the article and patient follow up; Dr H.J.: makes hematological section, literature search and patient follow up; Dr A.S.: writes the introduction section and article corrections; Dr F.H.: writes the case presentation section and article corrections; Q.H.: makes nephrology section, article corrections, supervisor and follow up of the patient.

## Conflicts of interest disclosure

The author declares that they have no conflicts of interest regarding this study. The author declares that it has not been published elsewhere and that it has not been submitted simultaneously for publication elsewhere.

## Research registration unique identifying number (UIN)

1. Name of the registry: NA.
2. Unique Identifying number or registration ID: NA.
3. Hyperlink to your specific registration (must be publicly accessible and will be checked): NA.

## Guarantor

The corresponding author is the guarantor of this manuscript.

## Provenance and peer review

Not commissioned, externally peer-reviewed.
